# Chest wall swelling and pneumothorax after shoulder arthroscopy: Were the 2 events totally independent?

**DOI:** 10.1097/MD.0000000000007020

**Published:** 2017-05-26

**Authors:** Jong Bun Kim, Min Kyung Choi, Yong Kyoung Jeon, Jae Myeong Lee

**Affiliations:** Department of Anesthesiology and Pain Medicine, Uijeongbu St. Mary's Hospital, College of Medicine, The Catholic University of Korea, Republic of Korea.

**Keywords:** arthroscopic shoulder surgery, chest wall edema, pneumothorax

## Abstract

**Rationale:**

Arthroscopic shoulder surgery (ASS) is a low mortality and morbidity procedure, but anesthetic complications are reported. There have been no reports of combined chest wall swelling and pneumothorax after ASS.

**Patient concerns:**

The patient's right lung was severely collapsed and the mediastinum was deviated after ASS.

**Diagnosis:**

Pneumothorax on right chest.

**Interventions:**

A chest tube was inserted and oxygen therapy was performed.

**Outcomes:**

The patient was discharged uneventfully.

**Lessons:**

Elective ASS has low mortality and morbidity rates, but we should be more concerned over the complications after ASS.

## 1 Introduction

Chest wall and neck swelling after arthroscopic shoulder surgery (ASS) occurs frequently, but pneumothorax after ASS has been rarely reported.^[1]^ Postoperatively, right chest wall and neck swelling was detected and an X-ray revealed severe right lung collapse and a deviated mediastinum.

## 2 Case report

**Figure 1:**
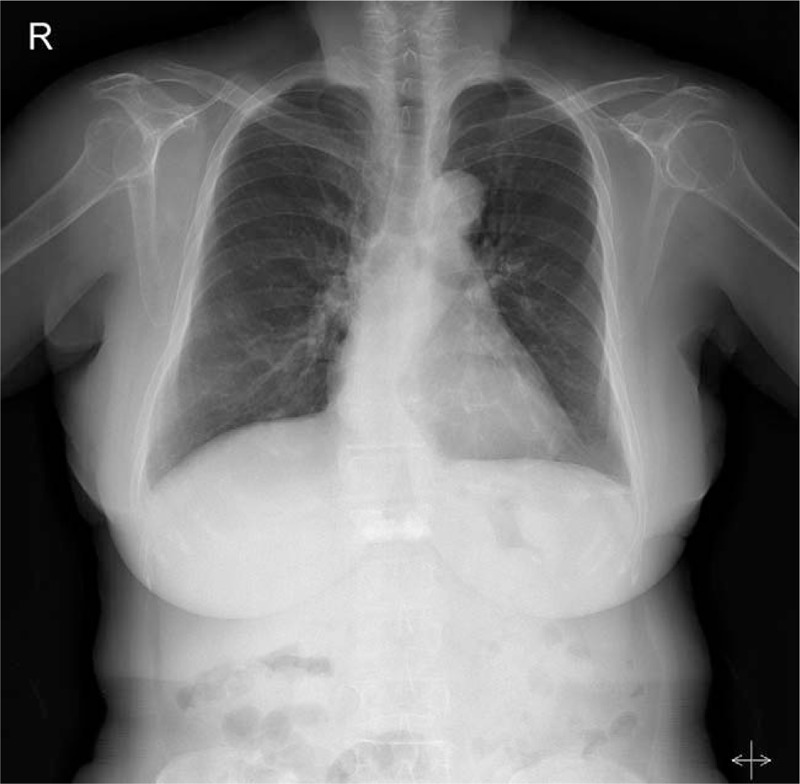
Preoperative chest X-ray shows no specific lesion.

This case was approved by the institutional review board of Uijeongbu St. Mary's Hospital of Catholic University of Korea. A 146 cm, 51 kg, 75-year-old woman was scheduled for ASS for subscapularis repair. She had taken medication for hypertension but had no other medical illness or a previous anesthetic history. She does not smoke and walks for 1 h everyday for her health. Her preoperative laboratory findings were within normal ranges, and chest X-ray showed no specific lesion (Fig. 1). Pulmonary function tests revealed a mild obstructive pattern, but she did not have any specific respiratory symptoms.

**Figure 2:**
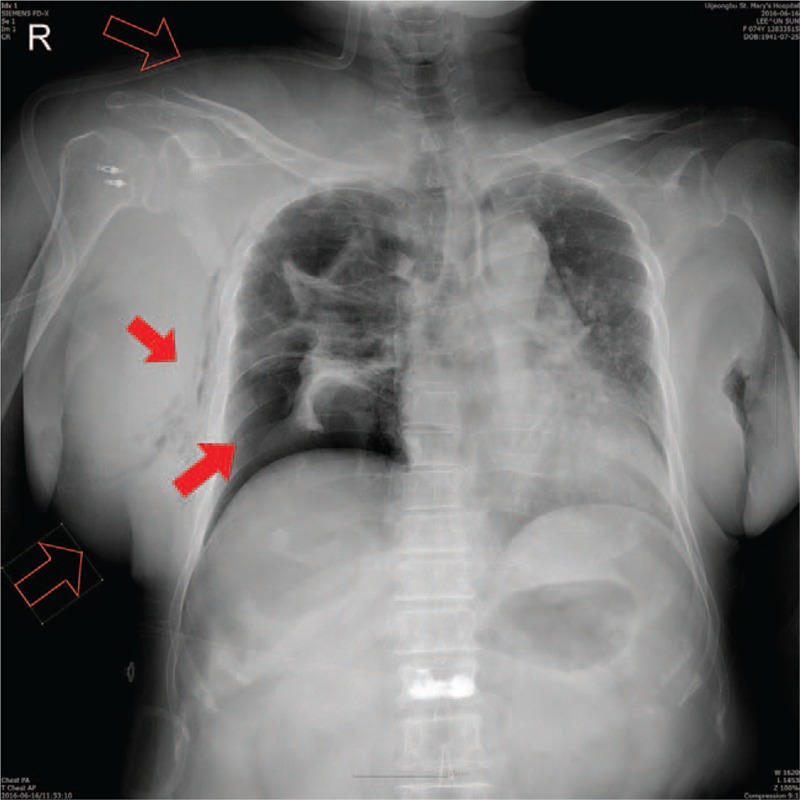
Neck and chest wall swelling (open arrows), and severe lung collapse and subcutaneous emphysema (closed arrows).

In the operating theater, electrocardiogram, blood pressure (BP), pulse oximetry (SPO_2_), and capnography were connected. Initial BP was 136/84 mm Hg, heart rate (HR) 71/min, and body temperature 36.3°C, but, initial SPO_2_ was 94%. General anesthesia was induced with propofol, remifentanil, and rocuronium, and the patient was intubated with a cuffed endotracheal tube of 7 mm internal diameter and fixed at 21 cm at the lips. Both lung sounds were clear. Anesthesia was maintained by air–O_2_–desflurane mixture and remifentanil infusion. The patient was ventilated with a tidal volume of 450 mL and peak end-expiratory pressure of 5 cm H_2_O, respiratory rate of 13/min, and airway pressure of 20 cm H_2_O. Intraoperative vital signs were stable, and SPO_2_ was 100% and airway pressure remained unchanged. She underwent arthroscopic subscapularis muscle repair and biceps tenotomy; the total anesthetic time was 2 h 40 min and surgery ended uneventfully. However, after opening the surgical drape, the right chest wall and neck were found to be severely swollen. The patient was breathing well and showed no airway obstructions, and so was extubated. In the postanesthesia care unit, BP was 130/80 mm Hg, HR 73/min, and respiratory rate 16 /min; however, SPO_2_ was 92% despite use of an O_2_ 5L/min oxygen mask. In the auscultation, right breathing sound could not be discerned, but she had no dyspnea and looked well. A chest X-ray showed that the right lung was collapsed (Fig. 2), subcutaneous emphysema was revealed and the mediastinum was deviated to the left. A chest tube was inserted into the right thorax and oxygen therapy was performed for 4 days. She remained in hospital for a further 2 days and was then discharged uneventfully. She refused to undergo check-up by chest computed tomography (CT).

## 3 Discussion

We often encounter chest wall swelling because of irrigating fluid extravasation after arthroscopic shoulder surgeries. Intermittently, the edema even extends to the neck and causes airway compression. Our patient had a low oxygen saturation preoperatively, but no specific disease or respiratory symptoms and so surgery was performed. After surgery, her right chest wall and neck were swollen but she did not complain of dyspnea and was breathing well. The decreased right lung sound was thought to be due the chest wall edema. We were concerned over the occurrence of hydrothorax for fear of extravasation of irrigating fluid into the thorax. The pneumothorax and subcutaneous emphysema were only detected upon examination of the chest X-ray. The mediastinum was deviated to the left. If the postoperative chest X-ray had been missed, a fatal event would likely have occurred. Intraoperative airway pressure elevation was not detected and intraoperative oxygen saturation was maintained.

ASS has some surgical and anesthetic complications. Anesthesia-related complications are air embolism,^[2]^ decreased cerebral oxygenation,^[3]^ quadriplegia in the sitting position,^[4]^ epinephrine induced arrhythmia,^[5]^ negative pressure pulmonary edema,^[6]^ airway complications, and pneumothorax.^[7]^ Pneumothorax and subcutaneous emphysema are rarely reported.^[1,8,9]^ In a previous study of pneumothorax in shoulder arthroscopy, the patients had risk factors such as respiratory disease or smoking history.^[1]^ Our patients did not have respiratory diseases such as asthma, chronic obstructive pulmonary disease, lung bullae; did not smoke; and had no chest X-ray abnormalities. The patient had refused a postoperative chest CT, but simple X-ray showed no lung parenchymal lesions. In another case report, the patient had no respiratory risk factors and the authors insisted that the arthroscopic pump pressure and shaving devices were the causes of the complications.^[9]^

In our patients, irrigating fluid was extravasated and chest wall and neck swelling was severe. We were concerned over the occurrence of hydrothorax combined with severe chest wall edema, but, occurrence of pneumothorax was an unexpected one. Pneumothorax together with chest wall swelling has not been reported to date, but we believe them to be relevant. High irrigating fluid pressure and volume during arthroscopy resulted in disruption of the connective tissue and caused chest wall swelling. Indeed, transmitted pressure and the force of the shaving device might have resulted in pneumothorax in the intrathorax. Both the high pressure resulting from irrigating fluid, shaving device, and entrained air might have contributed to the pneumothorax and subcutaneous emphysema. Careful use of arthroscopic pumps and suction devices and minimizing tissue air entrainment during shoulder surgery have been recommended.^[10]^ The amount of extravasated fluid depends on the procedure time, arthroscope pressure, patient position, and patient body weight.^[11]^

Regional anesthesia is performed frequently in shoulder surgery, and complications are reported even using ultrasound. Inadvertent puncture of the fascia or direct lung injury during block might have resulted in pneumothorax.^[12,13]^ Ultrasound does not completely prevent pneumothorax. We did not perform the any kind of brachial plexus block to this patient. If regional anesthesia had been applied, it would likely have been regarded as responsible for the pneumothorax.^[14]^ This case reinforces the notion that regional anesthesia is not only the cause of pneumothorax after ASS.

We misdiagnosed the postoperative low oxygen saturation being due to low preoperative saturation, obesity, and severe chest swelling, and we should have focused attention on the patient. Obese patients are considered have low saturation perioperatively and so other causes of low saturation could be missed. We have experienced numerous cases of neck and chest wall swelling after arthroscopy, but until now we tended to ignore the swelling and did not routinely check the X-rays. We now that in cases with a prolonged procedure duration, chest and neck swelling, and a low SPO_2_ after ASS, chest X-ray should be checked postoperatively.

Elective ASS has low mortality and morbidity rates, and most patients are in good health. Therefore, the physician and patient do not tend to worry about the complications. We should be more concerned over the complications that can occur after ASS.
